# Local production of alcohol based handrub solution (ABHS) in Liberia during the Ebola outbreak

**DOI:** 10.1186/2047-2994-4-S1-P8

**Published:** 2015-06-16

**Authors:** H Olivier, T Tarty Tyee, J Mulbah, M Massaquoi, B Dahn, L Bengaly, P Bonnabry, L Cingria, MB Matthey- Khouiti, S Weber, L Riegger, A Deuble, S Wallis, P Comtesse, D Pittet

**Affiliations:** 1 University Hospitals Geneva, Geneva, Switzerland

## Introduction

The hands transmit the major part of the infection. In addition of a low level of awareness of the Infection Prevention and Control (IPC), the lack of availability of the ABHS is usual.

## Objectives

The challenges were:

Register the product at the Ministry of Health and Social Welfare (MoHSW), provide the ABHS’s kits and the consumables, Train Liberian hospital’s pharmacist , select pilot hospitals with the support of the MoHSW, produce locally ABHS based on the WHO formula, evaluate the project and to ensure sustainability.

## Methods

The MoHSW has recorded the ABHS as part of the pharmaceutical product. Swiss Agency for Development and Cooperation (SDC) has provided 10 kits for the production of ABHS in addition of local provision of ethanol 95%. University Hospitals of Geneva is providing technical support.

The MoHSW has selected 3 following pilot hospitals: Redemption Hospital, Monrovia, James N. Davis Jr. Memorial Hospital (JDJ), Monrovia, Phebe Hospital N’Bonga.

In November 2014, 21 pharmacists and 1 laboratory technician were trained during 2 days, training that was given 2 times (10 persons/session). After the training, the production of the ABHS began in the 3 pilot hospitals.

## Results

### Monitoring & evaluation criteria

The monitoring and evaluation were done based on a site’s visit and a questionnaire.

The criteria used cover different aspects (logistic, production, distribution, effective use of the ABHS, inter-action with hospital management and the MoHSW). Interview with pharmacists, hospital staff and administration, and other agencies involved.

### Effective production & distribution

**Table 1:** 

**Redemption Hospital**	**1180 bottles produced**	**486 distributed**
**JDJ Hospital**	**1000 bottles produced**	**400 distributed**
**Phebe Hospital**	**1139 bottles produced**	**355 distributed**

The ABHS pharmacists spend 80% of their time on their regular work during a production week.

ABHS is produced according to the WHO standards.

In all three hospitals, 90% of the staff approached knew about the ABHS and used it.

## Conclusion

Despite the Ebola outbreak, this project has shown that it is possible to produce locally AHBS.

The findings of the Monitoring & Evaluation should lead to the selection of 7 additional health facilities for the distribution of the 7 remaining ABHS kits and the ethanol.

## Disclosure of interest

None declared.

